# Propane-1,2-diaminium bis­(pyridine-2,6-dicarboxyl­ato-κ^3^ *O* ^2^,*N*,*O* ^6^)cuprate(II) tetra­hydrate

**DOI:** 10.1107/S1600536811024378

**Published:** 2011-06-30

**Authors:** Hossein Aghabozorg, Ali Akbar Agah, Behrouz Notash, Masoud Mirzaei

**Affiliations:** a Faculty of Chemistry, Tarbiat Moallem University, 15614, Tehran, Iran; b Department of Chemistry, Shahid Beheshti University, G. C., Evin, Tehran, Iran; c Department of Chemistry, Ferdowsi University of Mashhad, Mashhad 917751436, Iran

## Abstract

In the title compound, (C_3_H_12_N_2_)[Cu(C_7_H_3_NO_4_)_2_]·4H_2_O, the Cu^II^ atom is six-coordinated in a distorted octa­hedral geometry by two tridentate pyridine-2,6-dicarboxyl­ate (pydc) ligands. In the crystal, inter­molecular O—H⋯O, N—H⋯O and weak C—H⋯O hydrogen bonds, as well as π–π stacking inter­actions between the pyridine rings of the pydc ligands [centroid–centroid distance = 3.4714 (14) Å] are present. C=O⋯π inter­actions between the carbonyl groups and pyridine rings [O⋯centroid distances = 3.150 (2) and 3.2233 (19) Å] are also observed.

## Related literature

For background to proton-transfer compounds, see: Aghabozorg *et al.* (2008*d* ▶). For related structures, see: Aghabozorg *et al.* (2008*a* ▶,*b* ▶,*c* ▶).
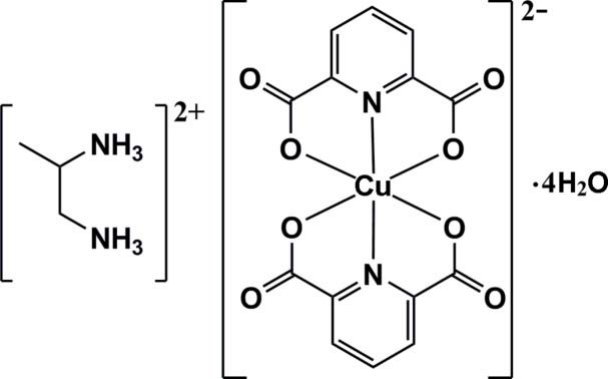


## Experimental

### 

#### Crystal data

(C_3_H_12_N_2_)[Cu(C_7_H_3_NO_4_)_2_]·4H_2_O
*M* *_r_* = 541.97
Orthorhombic, 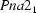

*a* = 20.919 (4) Å
*b* = 8.2015 (16) Å
*c* = 12.739 (3) Å
*V* = 2185.6 (7) Å^3^
*Z* = 4
Mo *K*α radiation
μ = 1.07 mm^−1^
*T* = 120 K
0.50 × 0.40 × 0.35 mm

#### Data collection

Stoe IPDS-2 diffractometer
Absorption correction: numerical (*X-SHAPE* and *X-RED32*; Stoe & Cie, 2005 ▶) *T*_min_ = 0.602, *T*_max_ = 0.684
9693 measured reflections
5040 independent reflections
4803 reflections with *I* > 2σ(*I*)
*R*_int_ = 0.029

#### Refinement

*R*[*F* ^2^ > 2σ(*F* ^2^)] = 0.028
*wR*(*F* ^2^) = 0.075
*S* = 1.09
5040 reflections
350 parameters
7 restraints
H atoms treated by a mixture of independent and constrained refinement
Δρ_max_ = 0.43 e Å^−3^
Δρ_min_ = −0.52 e Å^−3^
Absolute structure: Flack (1983 ▶), 1969 Friedel pairs
Flack parameter: −0.001 (10)

### 

Data collection: *X-AREA* (Stoe & Cie, 2005 ▶); cell refinement: *X-AREA*; data reduction: *X-AREA*; program(s) used to solve structure: *SHELXS97* (Sheldrick, 2008 ▶); program(s) used to refine structure: *SHELXL97* (Sheldrick, 2008 ▶); molecular graphics: *ORTEP-3* (Farrugia, 1997 ▶); software used to prepare material for publication: *WinGX* (Farrugia, 1999 ▶).

## Supplementary Material

Crystal structure: contains datablock(s) I, global. DOI: 10.1107/S1600536811024378/hy2413sup1.cif

Structure factors: contains datablock(s) I. DOI: 10.1107/S1600536811024378/hy2413Isup2.hkl

Additional supplementary materials:  crystallographic information; 3D view; checkCIF report

## Figures and Tables

**Table 1:** Hydrogen-bond geometry (Å, °)

*D*—H⋯*A*	*D*—H	H⋯*A*	*D*⋯*A*	*D*—H⋯*A*
C10—H10⋯O11^i^	0.93	2.59	3.476 (3)	160
C11—H11⋯O7^ii^	0.93	2.56	3.301 (3)	137
C15—H15*A*⋯O8^iii^	0.97	2.30	3.245 (3)	165
C16—H16⋯O5^iv^	0.98	2.53	3.321 (3)	138
N3—H3*A*⋯O6^iv^	0.89 (4)	1.93 (4)	2.812 (3)	170 (3)
N3—H3*B*⋯O11	0.95 (4)	1.88 (4)	2.773 (3)	155 (3)
N3—H3*C*⋯O2	0.90 (4)	1.91 (4)	2.794 (2)	167 (4)
N4—H4*A*⋯O10^iv^	0.86 (3)	1.94 (3)	2.786 (3)	165 (3)
N4—H4*B*⋯O12	0.83 (4)	2.00 (4)	2.811 (3)	165 (3)
N4—H4*C*⋯O4^v^	0.84 (2)	2.01 (2)	2.829 (3)	167 (3)
O9—H9*A*⋯O1	0.84 (2)	1.93 (2)	2.739 (3)	163 (3)
O9—H9*B*⋯O4^vi^	0.82 (2)	2.04 (2)	2.826 (3)	160 (3)
O10—H10*A*⋯O9	0.78 (4)	1.97 (4)	2.731 (3)	164 (4)
O10—H10*B*⋯O8^v^	0.85 (4)	1.88 (4)	2.724 (3)	170 (3)
O11—H11*A*⋯O3^v^	0.82 (2)	2.41 (3)	3.080 (2)	140 (3)
O11—H11*A*⋯O7^v^	0.82 (2)	2.30 (3)	2.957 (2)	138 (3)
O11—H11*B*⋯O10	0.82 (4)	1.98 (4)	2.781 (3)	169 (3)
O12—H12*A*⋯O2^vii^	0.79 (2)	1.99 (2)	2.770 (3)	170 (3)
O12—H12*B*⋯O6^iv^	0.81 (2)	2.09 (3)	2.786 (2)	144 (3)

Symmetry codes: (i) 
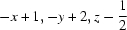
; (ii) 
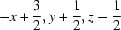
; (iii) 
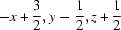
; (iv) 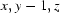; (v) 
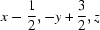
; (vi) 
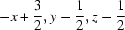
; (vii) 
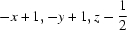
.

**Fig. 1.**
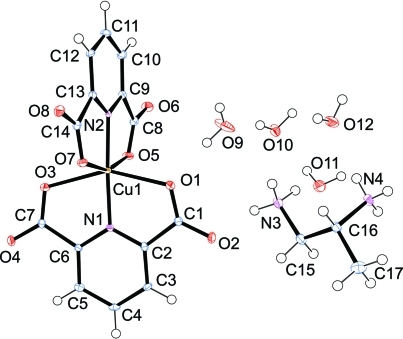
Molecular structure of the title compound, with displacement ellipsoids drawn at the 50% probability level.

**Fig. 2.**
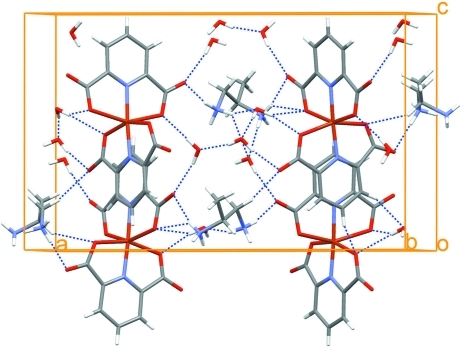
The packing diagram of the title compound, showing intermolecular N—H···O, O—H···O and weak C—H···O hydrogen bonds (blue dashed lines).

**Fig. 3.**
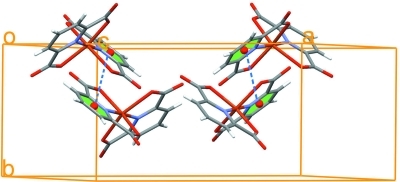
The packing diagram of the title compound, showing intermolecular π–π interactions [dashed lines, centroid–centroid distance = 3.4714 (14) Å]. Water molecules and cations have been omitted for clarity.

**Fig. 4.**
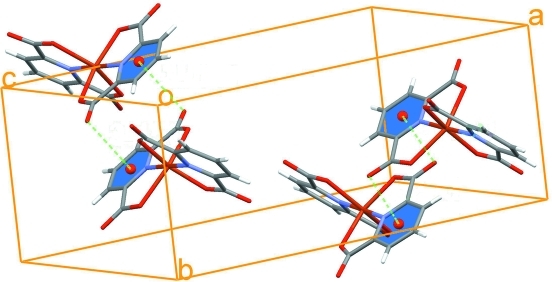
The packing diagram of the title compound, showing C═O···π interactions (dashed lines) between the pyridine rings and the carbonyl groups [O···centroid distances = 3.150 (2) and 3.2233 (19) Å]. Water molecules and cations have been omitted for clarity.

## supplementary crystallographic information

### Comment

Our group has previously reported some proton-transfer systems (Aghabozorg *et al.*, 2008*d*), using pyridine-2,6-dicarboxylic acid (pydcH_2_), propane-1,2-diamine (p-1,2-da) and propane-1,3-diamine (p-1,3-da), which formed the proton-transfer compounds (p-1,2-daH_2_)(pydcH)_2_.2H_2_O (Aghabozorg *et al.*, 2008*c*), (p-1,2-daH_2_)[Ni(pydc)_2_].4H_2_O (Aghabozorg *et al.*, 2008*b*), (p-1,3-daH_2_)[Cd(pydc)_2_].3.5H_2_O, (p-1,3-daH_2_)[Cu(pydc)_2_].4H_2_O and (p-1,3-daH2)[Co(pydc)_2_].4H_2_O (Aghabozorg *et al.*, 2008*a*).

We describe here the crystal structure of the title compound (Fig. 1). In the complex anion, the Cu^II^ ion is six-coordinated by two (pydc)^2-^ ligands in a distorted octahedral geometry. In the crystal, there are N—H···O, O—H···O and weak C—H···O intermolecular hydrogen bonds (Table 1, Fig. 2). There are also π–π stacking interactions between the pyridine rings of the pydc ligands [centroid–centroid distance = 3.4714 (14) Å], as shown in Fig. 3. In addition, there are C═O···π interactions (Fig. 4) between the carbonyl groups and pyridine rings [O···centroid distances = 3.150 (2) and 3.2233 (19) Å].

### Experimental

By mixing Cu(OAc)_2_.H_2_O (1 mmol), pyridine-2,6-dicarboxylic acid (2 mmol) and propane-1,2-diamine (1 mmol) in 20 ml water, a blue solution was obtained. Blue crystals of the title compound were obtained by allowing the mixture to stand at room temperature for a week.

### Refinement

H atoms of the protonated N atoms and water molecules were found in a difference Fourier map and their coordinates were refined and *U*_iso_ values were fixed, in which H4C, H9A, H9B, H11A, H12A and H12B were refined with distance restraints of N—H/O—H = 0.84 (2), 0.84 (2), 0.82 (2), 0.82 (2), 0.79 (2), 0.81 (2) Å. H atoms on C atoms were positioned geometrically and refined as riding atoms, with C—H = 0.93 (aromatic), 0.96 (CH_3_), 0.97 (CH_2_) and 0.98 (CH)Å and with *U*_iso_(H) = 1.2*U*_eq_(C).

### Crystal data

**Table:**

(C_3_H_12_N_2_)[Cu(C_7_H_3_NO_4_)_2_]·4H_2_O	*F*(000) = 1124
*M**_r_* = 541.97	*D*_x_ = 1.647 Mg m^−^^3^
Orthorhombic, *P**n**a*2_1_	Mo *K*α radiation, λ = 0.71073 Å
Hall symbol: P 2c -2n	Cell parameters from 5040 reflections
*a* = 20.919 (4) Å	θ = 2.5–29.1°
*b* = 8.2015 (16) Å	µ = 1.07 mm^−^^1^
*c* = 12.739 (3) Å	*T* = 120 K
*V* = 2185.6 (7) Å^3^	Block, blue
*Z* = 4	0.50 × 0.40 × 0.35 mm

### Data collection

**Table:**

Stoe IPDS-2 diffractometer	5040 independent reflections
Radiation source: fine-focus sealed tube	4803 reflections with *I* > 2σ(*I*)
graphite	*R*_int_ = 0.029
ω scans	θ_max_ = 29.1°, θ_min_ = 2.5°
Absorption correction: numerical (*X-SHAPE* and *X-RED32*; Stoe & Cie, 2005)	*h* = −24→28
*T*_min_ = 0.602, *T*_max_ = 0.684	*k* = −9→11
9693 measured reflections	*l* = −14→17

### Refinement

**Table:**

Refinement on *F*^2^	Secondary atom site location: difference Fourier map
Least-squares matrix: full	Hydrogen site location: inferred from neighbouring sites
*R*[*F*^2^ > 2σ(*F*^2^)] = 0.028	H atoms treated by a mixture of independent and constrained refinement
*wR*(*F*^2^) = 0.075	*w* = 1/[σ^2^(*F*_o_^2^) + (0.0422*P*)^2^ + 0.9494*P*] where *P* = (*F*_o_^2^ + 2*F*_c_^2^)/3
*S* = 1.09	(Δ/σ)_max_ = 0.003
5040 reflections	Δρ_max_ = 0.43 e Å^−^^3^
350 parameters	Δρ_min_ = −0.52 e Å^−^^3^
7 restraints	Absolute structure: Flack (1983), 1969 Friedel pairs
Primary atom site location: structure-invariant direct methods	Flack parameter: −0.001 (10)

### Fractional atomic coordinates and isotropic or equivalent isotropic displacement parameters (Å^2^)

**Table:**

	*x*	*y*	*z*	*U*_iso_*/*U*_eq_	
Cu1	0.730311 (10)	0.99084 (3)	0.53964 (3)	0.00819 (6)	
O1	0.65641 (8)	0.7928 (2)	0.57097 (13)	0.0156 (3)	
O2	0.60721 (7)	0.67722 (19)	0.70796 (13)	0.0133 (3)	
O3	0.81368 (7)	1.15817 (19)	0.58997 (13)	0.0121 (3)	
O4	0.86763 (7)	1.22026 (19)	0.73603 (13)	0.0126 (3)	
O5	0.65879 (7)	1.16886 (17)	0.53346 (15)	0.0134 (3)	
O6	0.59524 (8)	1.2936 (2)	0.41726 (14)	0.0151 (3)	
O7	0.80165 (7)	0.83190 (19)	0.48615 (13)	0.0109 (3)	
O8	0.85015 (8)	0.7555 (2)	0.33667 (13)	0.0129 (3)	
O9	0.56195 (10)	0.7606 (4)	0.42357 (17)	0.0426 (7)	
O10	0.44108 (9)	0.8652 (2)	0.46858 (15)	0.0162 (3)	
O11	0.42267 (8)	0.5941 (2)	0.59618 (14)	0.0171 (3)	
O12	0.46500 (9)	0.3499 (2)	0.38944 (16)	0.0216 (4)	
N1	0.73387 (8)	0.9634 (2)	0.69332 (17)	0.0079 (3)	
N2	0.72136 (9)	1.0156 (2)	0.38998 (18)	0.0077 (4)	
N3	0.54048 (9)	0.4390 (2)	0.59580 (16)	0.0124 (3)	
N4	0.44690 (9)	0.1638 (2)	0.57235 (16)	0.0116 (4)	
C1	0.64760 (10)	0.7709 (3)	0.66754 (17)	0.0096 (4)	
C2	0.69142 (10)	0.8648 (2)	0.74089 (18)	0.0088 (4)	
C3	0.69152 (10)	0.8437 (2)	0.84916 (18)	0.0094 (4)	
H3	0.6616	0.7763	0.8813	0.011*	
C4	0.73706 (10)	0.9252 (3)	0.90831 (18)	0.0112 (4)	
H4	0.7382	0.9122	0.9808	0.013*	
C5	0.78101 (11)	1.0265 (3)	0.85857 (19)	0.0099 (4)	
H5	0.8121	1.0813	0.8971	0.012*	
C6	0.77765 (10)	1.0443 (2)	0.75039 (18)	0.0079 (4)	
C7	0.82336 (9)	1.1500 (2)	0.68727 (18)	0.0089 (4)	
C8	0.63972 (10)	1.1994 (3)	0.44162 (19)	0.0109 (4)	
C9	0.67591 (10)	1.1157 (2)	0.35435 (18)	0.0093 (4)	
C10	0.66963 (11)	1.1435 (2)	0.24765 (18)	0.0110 (4)	
H10	0.6379	1.2123	0.2221	0.013*	
C11	0.71196 (11)	1.0660 (3)	0.17933 (17)	0.0118 (4)	
H11	0.7089	1.0839	0.1074	0.014*	
C12	0.75891 (11)	0.9616 (3)	0.21866 (18)	0.0097 (4)	
H12	0.7875	0.9093	0.1739	0.012*	
C13	0.76179 (10)	0.9379 (3)	0.32665 (17)	0.0090 (4)	
C14	0.80893 (10)	0.8320 (2)	0.38701 (19)	0.0097 (4)	
C15	0.53174 (11)	0.3070 (3)	0.67483 (19)	0.0135 (4)	
H15B	0.5001	0.3411	0.7259	0.016*	
H15A	0.5717	0.2899	0.7117	0.016*	
C16	0.51050 (10)	0.1466 (2)	0.62550 (18)	0.0114 (4)	
H16	0.5423	0.1141	0.5730	0.014*	
C17	0.50603 (13)	0.0132 (3)	0.7083 (2)	0.0188 (5)	
H17B	0.4754	0.0440	0.7607	0.023*	
H17C	0.5471	−0.0017	0.7406	0.023*	
H17A	0.4928	−0.0869	0.6758	0.023*	
H3A	0.5597 (15)	0.404 (4)	0.538 (3)	0.023*	
H4A	0.4377 (15)	0.074 (4)	0.540 (3)	0.023*	
H9A	0.5887 (14)	0.790 (4)	0.468 (2)	0.023*	
H10A	0.4750 (18)	0.846 (4)	0.446 (3)	0.023*	
H11A	0.3889 (11)	0.562 (4)	0.572 (3)	0.023*	
H12A	0.4484 (15)	0.340 (4)	0.3341 (18)	0.023*	
H3B	0.4992 (17)	0.477 (4)	0.577 (3)	0.023*	
H4B	0.4502 (15)	0.233 (4)	0.525 (3)	0.023*	
H9B	0.5786 (16)	0.726 (4)	0.3696 (19)	0.023*	
H10B	0.4158 (17)	0.830 (4)	0.421 (3)	0.023*	
H11B	0.4249 (16)	0.680 (4)	0.564 (3)	0.023*	
H12B	0.4996 (11)	0.316 (4)	0.372 (3)	0.023*	
H3C	0.5641 (17)	0.520 (4)	0.622 (3)	0.023*	
H4C	0.4190 (13)	0.200 (4)	0.613 (2)	0.023*	

### Atomic displacement parameters (Å^2^)

**Table:**

	*U*^11^	*U*^22^	*U*^33^	*U*^12^	*U*^13^	*U*^23^
Cu1	0.00687 (10)	0.01264 (10)	0.00506 (10)	0.00041 (8)	−0.00005 (12)	0.00104 (10)
O1	0.0129 (7)	0.0252 (8)	0.0088 (8)	−0.0069 (6)	−0.0014 (6)	−0.0002 (6)
O2	0.0105 (7)	0.0159 (7)	0.0134 (8)	−0.0052 (5)	0.0017 (6)	−0.0014 (6)
O3	0.0133 (7)	0.0152 (7)	0.0079 (8)	−0.0031 (5)	−0.0008 (6)	0.0008 (6)
O4	0.0088 (7)	0.0169 (7)	0.0122 (8)	−0.0040 (6)	−0.0002 (6)	−0.0021 (6)
O5	0.0113 (6)	0.0217 (7)	0.0072 (7)	0.0036 (5)	0.0009 (7)	0.0002 (7)
O6	0.0115 (7)	0.0202 (8)	0.0135 (8)	0.0066 (6)	−0.0001 (6)	0.0019 (6)
O7	0.0096 (7)	0.0150 (7)	0.0081 (8)	0.0023 (5)	0.0003 (6)	0.0009 (5)
O8	0.0090 (7)	0.0178 (7)	0.0120 (8)	0.0024 (6)	0.0027 (6)	0.0000 (6)
O9	0.0142 (9)	0.100 (2)	0.0136 (10)	0.0126 (11)	−0.0035 (8)	−0.0176 (12)
O10	0.0133 (8)	0.0181 (7)	0.0172 (9)	−0.0011 (6)	−0.0005 (7)	−0.0052 (6)
O11	0.0118 (7)	0.0214 (8)	0.0180 (9)	0.0024 (6)	−0.0011 (7)	0.0026 (7)
O12	0.0146 (8)	0.0367 (10)	0.0135 (9)	0.0005 (7)	−0.0046 (7)	0.0009 (7)
N1	0.0060 (8)	0.0104 (8)	0.0073 (9)	0.0005 (6)	−0.0002 (7)	0.0003 (8)
N2	0.0049 (7)	0.0120 (8)	0.0063 (9)	−0.0024 (6)	−0.0006 (7)	0.0007 (7)
N3	0.0096 (8)	0.0148 (8)	0.0128 (9)	−0.0006 (6)	−0.0004 (7)	−0.0032 (7)
N4	0.0073 (7)	0.0147 (8)	0.0128 (9)	0.0011 (6)	−0.0003 (7)	−0.0017 (7)
C1	0.0069 (8)	0.0116 (9)	0.0104 (10)	0.0006 (7)	−0.0001 (8)	0.0003 (7)
C2	0.0062 (8)	0.0097 (8)	0.0104 (10)	0.0007 (7)	−0.0001 (8)	−0.0002 (7)
C3	0.0093 (9)	0.0114 (9)	0.0074 (9)	0.0006 (7)	0.0025 (8)	0.0023 (7)
C4	0.0146 (10)	0.0142 (9)	0.0050 (9)	0.0024 (8)	0.0014 (8)	−0.0005 (7)
C5	0.0112 (9)	0.0101 (8)	0.0085 (11)	0.0025 (7)	−0.0005 (8)	−0.0015 (7)
C6	0.0073 (8)	0.0098 (8)	0.0066 (10)	0.0007 (7)	−0.0006 (7)	−0.0004 (7)
C7	0.0064 (8)	0.0090 (8)	0.0113 (10)	0.0007 (6)	0.0007 (8)	−0.0001 (7)
C8	0.0088 (9)	0.0132 (8)	0.0107 (10)	0.0000 (7)	0.0022 (8)	−0.0002 (7)
C9	0.0076 (9)	0.0117 (9)	0.0085 (9)	−0.0009 (7)	−0.0007 (8)	0.0006 (7)
C10	0.0125 (9)	0.0099 (8)	0.0105 (10)	−0.0009 (7)	0.0002 (8)	0.0009 (7)
C11	0.0156 (10)	0.0131 (9)	0.0066 (10)	−0.0028 (8)	−0.0009 (8)	−0.0004 (7)
C12	0.0116 (9)	0.0109 (8)	0.0065 (10)	−0.0032 (8)	0.0019 (8)	−0.0014 (7)
C13	0.0077 (9)	0.0097 (9)	0.0095 (11)	−0.0012 (7)	−0.0009 (8)	0.0021 (7)
C14	0.0107 (9)	0.0079 (8)	0.0105 (10)	−0.0032 (7)	−0.0005 (8)	0.0004 (7)
C15	0.0117 (9)	0.0175 (9)	0.0112 (10)	−0.0023 (8)	−0.0013 (8)	−0.0018 (8)
C16	0.0091 (9)	0.0144 (9)	0.0108 (10)	0.0000 (7)	−0.0008 (8)	−0.0015 (7)
C17	0.0220 (11)	0.0188 (10)	0.0156 (13)	0.0035 (8)	−0.0001 (10)	0.0023 (9)

### Geometric parameters (Å, °)

**Table:**

Cu1—N2	1.926 (2)	N4—C16	1.499 (3)
Cu1—N1	1.972 (2)	N4—H4A	0.86 (3)
Cu1—O5	2.0920 (14)	N4—H4B	0.83 (4)
Cu1—O7	2.0953 (16)	N4—H4C	0.84 (2)
Cu1—O1	2.2775 (17)	C1—C2	1.519 (3)
Cu1—O3	2.3100 (16)	C2—C3	1.390 (3)
O1—C1	1.257 (3)	C3—C4	1.386 (3)
O2—C1	1.253 (3)	C3—H3	0.9300
O3—C7	1.258 (3)	C4—C5	1.392 (3)
O4—C7	1.255 (3)	C4—H4	0.9300
O5—C8	1.261 (3)	C5—C6	1.387 (3)
O6—C8	1.248 (3)	C5—H5	0.9300
O7—C14	1.272 (3)	C6—C7	1.521 (3)
O8—C14	1.245 (3)	C8—C9	1.510 (3)
O9—H9A	0.84 (2)	C9—C10	1.384 (3)
O9—H9B	0.82 (2)	C10—C11	1.395 (3)
O10—H10A	0.78 (4)	C10—H10	0.9300
O10—H10B	0.85 (4)	C11—C12	1.396 (3)
O11—H11A	0.82 (2)	C11—H11	0.9300
O11—H11B	0.82 (4)	C12—C13	1.390 (3)
O12—H12A	0.79 (2)	C12—H12	0.9300
O12—H12B	0.81 (2)	C13—C14	1.523 (3)
N1—C6	1.344 (3)	C15—C16	1.524 (3)
N1—C2	1.345 (3)	C15—H15B	0.9700
N2—C13	1.331 (3)	C15—H15A	0.9700
N2—C9	1.336 (3)	C16—C17	1.523 (3)
N3—C15	1.490 (3)	C16—H16	0.9800
N3—H3A	0.89 (4)	C17—H17B	0.9600
N3—H3B	0.95 (4)	C17—H17C	0.9600
N3—H3C	0.90 (4)	C17—H17A	0.9600
			
N2—Cu1—N1	176.55 (8)	C3—C4—H4	120.2
N2—Cu1—O5	79.62 (8)	C5—C4—H4	120.2
N1—Cu1—O5	98.27 (8)	C6—C5—C4	118.8 (2)
N2—Cu1—O7	79.21 (8)	C6—C5—H5	120.6
N1—Cu1—O7	103.00 (7)	C4—C5—H5	120.6
O5—Cu1—O7	158.64 (7)	N1—C6—C5	121.3 (2)
N2—Cu1—O1	100.52 (7)	N1—C6—C7	115.1 (2)
N1—Cu1—O1	76.72 (7)	C5—C6—C7	123.6 (2)
O5—Cu1—O1	91.08 (6)	O4—C7—O3	125.6 (2)
O7—Cu1—O1	95.55 (6)	O4—C7—C6	117.6 (2)
N2—Cu1—O3	106.59 (7)	O3—C7—C6	116.74 (18)
N1—Cu1—O3	76.33 (7)	O6—C8—O5	126.1 (2)
O5—Cu1—O3	97.80 (6)	O6—C8—C9	118.2 (2)
O7—Cu1—O3	85.54 (6)	O5—C8—C9	115.73 (19)
O1—Cu1—O3	152.56 (7)	N2—C9—C10	120.2 (2)
C1—O1—Cu1	111.91 (14)	N2—C9—C8	112.7 (2)
C7—O3—Cu1	111.32 (13)	C10—C9—C8	126.90 (19)
C8—O5—Cu1	113.56 (15)	C9—C10—C11	118.5 (2)
C14—O7—Cu1	114.06 (14)	C9—C10—H10	120.7
H9A—O9—H9B	113 (3)	C11—C10—H10	120.7
H10A—O10—H10B	104 (3)	C10—C11—C12	120.1 (2)
H11A—O11—H11B	98 (3)	C10—C11—H11	119.9
H12A—O12—H12B	97 (4)	C12—C11—H11	119.9
C6—N1—C2	120.2 (2)	C13—C12—C11	118.1 (2)
C6—N1—Cu1	120.43 (15)	C13—C12—H12	120.9
C2—N1—Cu1	119.39 (16)	C11—C12—H12	120.9
C13—N2—C9	122.7 (2)	N2—C13—C12	120.4 (2)
C13—N2—Cu1	119.20 (17)	N2—C13—C14	112.2 (2)
C9—N2—Cu1	118.04 (17)	C12—C13—C14	127.39 (19)
C15—N3—H3A	113 (2)	O8—C14—O7	126.5 (2)
C15—N3—H3B	107 (2)	O8—C14—C13	118.4 (2)
H3A—N3—H3B	108 (3)	O7—C14—C13	115.11 (19)
C15—N3—H3C	111 (2)	N3—C15—C16	112.60 (19)
H3A—N3—H3C	108 (3)	N3—C15—H15B	109.1
H3B—N3—H3C	111 (3)	C16—C15—H15B	109.1
C16—N4—H4A	109 (2)	N3—C15—H15A	109.1
C16—N4—H4B	109 (2)	C16—C15—H15A	109.1
H4A—N4—H4B	105 (3)	H15B—C15—H15A	107.8
C16—N4—H4C	112 (2)	N4—C16—C17	109.01 (18)
H4A—N4—H4C	116 (3)	N4—C16—C15	111.34 (17)
H4B—N4—H4C	106 (3)	C17—C16—C15	110.6 (2)
O2—C1—O1	126.1 (2)	N4—C16—H16	108.6
O2—C1—C2	117.73 (19)	C17—C16—H16	108.6
O1—C1—C2	116.18 (19)	C15—C16—H16	108.6
N1—C2—C3	121.4 (2)	C16—C17—H17B	109.5
N1—C2—C1	115.23 (19)	C16—C17—H17C	109.5
C3—C2—C1	123.26 (19)	H17B—C17—H17C	109.5
C4—C3—C2	118.7 (2)	C16—C17—H17A	109.5
C4—C3—H3	120.6	H17B—C17—H17A	109.5
C2—C3—H3	120.6	H17C—C17—H17A	109.5
C3—C4—C5	119.6 (2)		
			
N2—Cu1—O1—C1	−171.17 (16)	O1—C1—C2—C3	−174.4 (2)
N1—Cu1—O1—C1	6.72 (15)	N1—C2—C3—C4	−0.8 (3)
O5—Cu1—O1—C1	−91.53 (16)	C1—C2—C3—C4	174.61 (18)
O7—Cu1—O1—C1	108.80 (15)	C2—C3—C4—C5	0.5 (3)
O3—Cu1—O1—C1	17.8 (2)	C3—C4—C5—C6	0.5 (3)
N2—Cu1—O3—C7	−179.84 (14)	C2—N1—C6—C5	1.1 (3)
N1—Cu1—O3—C7	2.05 (14)	Cu1—N1—C6—C5	−179.91 (16)
O5—Cu1—O3—C7	98.72 (14)	C2—N1—C6—C7	179.27 (18)
O7—Cu1—O3—C7	−102.50 (14)	Cu1—N1—C6—C7	−1.7 (3)
O1—Cu1—O3—C7	−9.0 (2)	C4—C5—C6—N1	−1.3 (3)
N2—Cu1—O5—C8	5.37 (15)	C4—C5—C6—C7	−179.37 (18)
N1—Cu1—O5—C8	−171.87 (15)	Cu1—O3—C7—O4	175.85 (16)
O7—Cu1—O5—C8	13.2 (3)	Cu1—O3—C7—C6	−3.5 (2)
O1—Cu1—O5—C8	−95.13 (15)	N1—C6—C7—O4	−175.73 (19)
O3—Cu1—O5—C8	110.91 (15)	C5—C6—C7—O4	2.4 (3)
N2—Cu1—O7—C14	4.09 (14)	N1—C6—C7—O3	3.6 (3)
N1—Cu1—O7—C14	−178.60 (14)	C5—C6—C7—O3	−178.2 (2)
O5—Cu1—O7—C14	−3.7 (3)	Cu1—O5—C8—O6	175.81 (18)
O1—Cu1—O7—C14	103.77 (14)	Cu1—O5—C8—C9	−5.7 (2)
O3—Cu1—O7—C14	−103.75 (15)	C13—N2—C9—C10	−0.5 (3)
O5—Cu1—N1—C6	−96.10 (17)	Cu1—N2—C9—C10	176.89 (15)
O7—Cu1—N1—C6	82.02 (18)	C13—N2—C9—C8	−175.35 (18)
O1—Cu1—N1—C6	174.75 (19)	Cu1—N2—C9—C8	2.1 (2)
O3—Cu1—N1—C6	−0.02 (16)	O6—C8—C9—N2	−178.67 (19)
O5—Cu1—N1—C2	82.93 (17)	O5—C8—C9—N2	2.7 (3)
O7—Cu1—N1—C2	−98.95 (17)	O6—C8—C9—C10	6.9 (3)
O1—Cu1—N1—C2	−6.22 (16)	O5—C8—C9—C10	−171.7 (2)
O3—Cu1—N1—C2	179.00 (18)	N2—C9—C10—C11	−0.5 (3)
O5—Cu1—N2—C13	173.65 (16)	C8—C9—C10—C11	173.5 (2)
O7—Cu1—N2—C13	−3.47 (15)	C9—C10—C11—C12	0.7 (3)
O1—Cu1—N2—C13	−97.18 (16)	C10—C11—C12—C13	0.1 (3)
O3—Cu1—N2—C13	78.52 (16)	C9—N2—C13—C12	1.4 (3)
O5—Cu1—N2—C9	−3.87 (15)	Cu1—N2—C13—C12	−176.00 (16)
O7—Cu1—N2—C9	179.02 (16)	C9—N2—C13—C14	179.81 (18)
O1—Cu1—N2—C9	85.30 (16)	Cu1—N2—C13—C14	2.4 (2)
O3—Cu1—N2—C9	−99.00 (16)	C11—C12—C13—N2	−1.2 (3)
Cu1—O1—C1—O2	175.48 (17)	C11—C12—C13—C14	−179.30 (19)
Cu1—O1—C1—C2	−6.0 (2)	Cu1—O7—C14—O8	175.91 (17)
C6—N1—C2—C3	0.0 (3)	Cu1—O7—C14—C13	−4.0 (2)
Cu1—N1—C2—C3	−179.01 (15)	N2—C13—C14—O8	−178.59 (18)
C6—N1—C2—C1	−175.74 (18)	C12—C13—C14—O8	−0.3 (3)
Cu1—N1—C2—C1	5.2 (2)	N2—C13—C14—O7	1.3 (3)
O2—C1—C2—N1	179.95 (19)	C12—C13—C14—O7	179.6 (2)
O1—C1—C2—N1	1.3 (3)	N3—C15—C16—N4	−61.9 (2)
O2—C1—C2—C3	4.3 (3)	N3—C15—C16—C17	176.67 (19)

### Hydrogen-bond geometry (Å, °)

**Table:**

*D*—H···*A*	*D*—H	H···*A*	*D*···*A*	*D*—H···*A*
C10—H10···O11^i^	0.93	2.59	3.476 (3)	160
C11—H11···O7^ii^	0.93	2.56	3.301 (3)	137
C15—H15A···O8^iii^	0.97	2.30	3.245 (3)	165
C16—H16···O5^iv^	0.98	2.53	3.321 (3)	138
N3—H3A···O6^iv^	0.89 (4)	1.93 (4)	2.812 (3)	170 (3)
N3—H3B···O11	0.95 (4)	1.88 (4)	2.773 (3)	155 (3)
N3—H3C···O2	0.90 (4)	1.91 (4)	2.794 (2)	167 (4)
N4—H4A···O10^iv^	0.86 (3)	1.94 (3)	2.786 (3)	165 (3)
N4—H4B···O12	0.83 (4)	2.00 (4)	2.811 (3)	165 (3)
N4—H4C···O4^v^	0.84 (2)	2.01 (2)	2.829 (3)	167 (3)
O9—H9A···O1	0.84 (2)	1.93 (2)	2.739 (3)	163 (3)
O9—H9B···O4^vi^	0.82 (2)	2.04 (2)	2.826 (3)	160 (3)
O10—H10A···O9	0.78 (4)	1.97 (4)	2.731 (3)	164 (4)
O10—H10B···O8^v^	0.85 (4)	1.88 (4)	2.724 (3)	170 (3)
O11—H11A···O3^v^	0.82 (2)	2.41 (3)	3.080 (2)	140 (3)
O11—H11A···O7^v^	0.82 (2)	2.30 (3)	2.957 (2)	138 (3)
O11—H11B···O10	0.82 (4)	1.98 (4)	2.781 (3)	169 (3)
O12—H12A···O2^vii^	0.79 (2)	1.99 (2)	2.770 (3)	170 (3)
O12—H12B···O6^iv^	0.81 (2)	2.09 (3)	2.786 (2)	144 (3)

Symmetry codes: (i) −*x*+1, −*y*+2, *z*−1/2; (ii) −*x*+3/2, *y*+1/2, *z*−1/2; (iii) −*x*+3/2, *y*−1/2, *z*+1/2; (iv) *x*, *y*−1, *z*; (v) *x*−1/2, −*y*+3/2, *z*; (vi) −*x*+3/2, *y*−1/2, *z*−1/2; (vii) −*x*+1, −*y*+1, *z*−1/2.

## References

[bb1] Aghabozorg, H., Ghadermazi, M., Nakhjavan, B. & Manteghi, F. (2008*a*). *J. Chem. Crystallogr.* **38**, 135–145.

[bb2] Aghabozorg, H., Heidari, M., Bagheri, S., Attar Gharamaleki, J. & Ghadermazi, M. (2008*b*). *Acta Cryst.* E**64**, m874–m875. 10.1107/S1600536808016309 PMC2961796 21202746

[bb3] Aghabozorg, H., Heidari, M., Ghadermazi, M. & Attar Gharamaleki, J. (2008*c*). *Acta Cryst.* E**64**, o1045–o1046. 10.1107/S1600536808013263 PMC2961371 21202565

[bb4] Aghabozorg, H., Manteghi, F. & Sheshmani, S. (2008*d*). *J. Iran. Chem. Soc.* **5**, 184–227.

[bb5] Farrugia, L. J. (1997). *J. Appl. Cryst.* **30**, 565.

[bb6] Farrugia, L. J. (1999). *J. Appl. Cryst.* **32**, 837–838.

[bb7] Flack, H. D. (1983). *Acta Cryst.* A**39**, 876–881.

[bb8] Sheldrick, G. M. (2008). *Acta Cryst.* A**64**, 112–122. 10.1107/S0108767307043930 18156677

[bb9] Stoe & Cie (2005). *X-AREA*, *X-SHAPE* and *X-RED32* Stoe & Cie, Darmstadt, Germany.

